# Sphingosine 1-phosphate receptors and sphingosine kinase 1: novel biomarkers for clinical prognosis in breast, prostate, and hematological cancers

**DOI:** 10.3389/fonc.2012.00168

**Published:** 2012-12-03

**Authors:** Susan Pyne, Joanne Edwards, Jan Ohotski, Nigel J. Pyne

**Affiliations:** ^1^Cell Biology Group, Strathclyde Institute of Pharmacy and Biomedical Sciences, University of StrathclydeGlasgow, UK; ^2^Unit of Experimental Therapeutics, Institute of Cancer, College of Medical, Veterinary and Life Sciences, University of GlasgowGlasgow, UK

**Keywords:** sphingosine 1-phosphate, triple negative breast cancer, estrogen receptor, disease-specific survival, recurrence

## Abstract

There is substantial evidence for a role in cancer of the bioactive lipid sphingosine 1-phosphate (S1P), the enzyme sphingosine kinase 1 (that catalyses S1P formation) and S1P-specific G protein-coupled receptors. This perspective highlights recent findings demonstrating that sphingosine kinase 1 and S1P receptors are new important biomarkers for detection of early cancer and progression to aggressive cancer. The impact of the sub-cellular distribution of S1P metabolizing enzymes and S1P receptors and their spatial functional interaction with oncogenes is considered with respect to prognostic outcome. These findings suggest that S1P, in addition to being a biomarker of clinical prognosis, might also be a new therapeutic target for intervention in cancer.

## INTRODUCTION

Effective cancer therapy remains an important medical challenge. Genetic instability that leads to constitutive activating mutations of oncogenes and inactivating mutations of tumor suppressors leads to the hallmarks of cancer (Hanahan and Weinberg, 2011). It is important to identify new biomarkers that report early stage cancer or early transformation to aggressive cancer and ideally from clinical specimens. This might therefore enable early detection of cancer and inform on appropriate personalized chemotherapeutic intervention, thereby affording opportunities for improved therapies. It also follows that these biomarkers can be used to enable monitoring of effective drug intervention specific to a personalized clinical approach. Sphingosine 1-phosphate (S1P) is a bioactive lipid that has emerged as having an important role in both solid tumors and hematological cancer (Pyne and Pyne, 2010). There is now evidence that the enzymes involved in the metabolism of S1P and the cellular targets of this signaling lipid are important new therapeutically relevant biomarkers of clinical prognosis. Indeed, S1P promotes neoplastic transformation, enhances cell survival/reduces apoptosis, induces chemotherapeutic resistance, reduces senescence, promotes angiogenesis and creation of a tumor microenvironment, increases invasiveness/metastasis, and is involved in inflammation (Pyne and Pyne, 2010).

## SPHINGOSINE 1-PHOSPHATE METABOLISM AND ACTION IN CANCER

Sphingosine is formed from breakdown of complex sphingolipids and is further metabolized to S1P by the enzyme, sphingosine kinase. There are two isoforms of sphingosine kinase (SK1 and SK2) that are encoded by distinct genes and differ in their biochemical properties, sub-cellular localization and functions (Pyne and Pyne, 2010). S1P can be irreversibly degraded by S1P lyase, which represents the only exit point in the sphingolipid metabolic pathway. Alternatively, S1P can be recycled to sphingosine by the action of lipid phosphate- and S1P-specific phosphatases. S1P is a bioactive lipid that binds to five different G protein-coupled S1P receptors (termed S1P_1-5_) to induce cellular responses. Intracellular S1P directly binds to TRAF2 to regulate RIP1/NFκB signaling (Alvarez et al., 2010), to prohibitin 2 to affect mitochondrial oxidative phosphorylation (Strub et al., 2011), and to β-site APP cleaving enzyme-1 to increase amyloid-β peptide production (Takasugi et al., 2011). So what is the relevance of S1P to cancer? A major finding that addresses this question is that SK1 mRNA transcript and/or SK1 protein expression are increased in various human tumors (Pyne and Pyne, 2010). Moreover, S1P binds to and inhibits HDAC1/2 activity to modulate histone acetylation leading to induction of immediate early genes (Hait et al., 2009). High SK1 expression in cancer cells confers positive selection to these cells, a consequence of a survival and growth advantage induced by over-expression of the enzyme. SK1 is also involved in the acquisition of replicative immortality. This is exemplified by the finding that genotoxic-induced expression of p53 in cancer cells leads to the down-regulation of SK1 expression and the induction of death by cellular senescence (Heffernan-Stroud et al., 2011). Moreover, SK1 can function as an oncogene as evidenced by the finding that over-expression of SK1 in fibroblasts induces their transformation to fibrosarcoma (Xia et al., 2000). Whereas knockdown of SK1 reduces proliferation of, for example, glioblastoma cells (Van Brocklyn et al., 2005) and androgen-independent PC-3 prostate cancer cells (Akao et al., 2006), larger, more vascularized, resistant tumors are formed when cancer cells over-expressing SK1 are injected or implanted into mice (Pyne and Pyne, 2010). In addition, SK1 is a cellular “sensor” and confers chemotherapeutic resistance as it can promote the survival of cancer cells in the presence of anti-cancer agents (Loveridge et al., 2010). Therefore, targeting SK1 offers new approaches to the development of novel anti-cancer therapies.

There is also evidence for a role in cancer of S1P lyase (SPL), the enzyme that catalyses cleavage of S1P into hexadecenal and phosphoethanolamine. For example, the level of *SPL* mRNA expression is reduced in intestinal metastatic tumors (Oskouian et al., 2006) and the sensitivity of lung cancer cells to cisplatin and doxorubicin is increased by over-expression of SPL (Min et al., 2005). In addition, SPL is down-regulated in colon cancer, while over-expression of the enzyme induces apoptosis in HEK 293 cells (Oskouian et al., 2006). The early diagnosis of cancer is a key medical need that enhances the chances of successful treatment. In this regard, SPL is down-regulated in benign adenoma lesions of the *Apc*^Min/+^ mouse model, with a concomitant increase in sphingosine levels, which induces cell death (Oskouian and Saba, 2007). Cancer progression and full transformation to malignant tumors might arise from a selective pressure to increase SK1 expression to remove sphingosine and thereby promote adenoma cell survival, which might also promote neoplastic conversion. This is an example where early detection of increased expression of SK1 in the benign tumor might warrant early chemotherapeutic intervention. This seems to be borne out as knockout of the SK1 gene in a mouse model of multiple intestinal adenoma (Apc^Min/+^) reduces intestinal adenoma size (Kohno et al., 2006). In addition, adenomas exhibit higher levels of SK1 compared with normal mucosa and colon cancer cells that have undergone metastasis have higher expression of SK1 compared with tumors without metastasis. SK1 levels are also elevated in the azoxymethane (AOM) murine model of colon cancer. In addition, S1P levels in the blood were higher in mice with colon cancers than in those without cancers (Kawamori et al., 2009).

There is also a substantial body of evidence to demonstrate a role for S1P receptors in cancer progression. For instance, S1P stimulates migration of fibrosarcoma cells through S1P_1_ (Fisher et al., 2006) and of gastric cancer cells through S1P_3_ (Yamashita et al., 2006). Therefore, targeting SK1 and S1P receptors is an attractive approach to producing new preclinical candidates for the treatment of cancer.

## SPHINGOSINE 1-PHOSPHATE AS A BIOMARKER FOR CANCER DETECTION

Recent studies have demonstrated that the circulating levels of S1P might serve as a biomarker for cancer progression. Pchejetski and colleagues have reported that circulating S1P levels are considerably lower in patients with prostate cancer compared with healthy patients and that this represents an early marker for progression to androgen-independence (Nunes et al., 2012). S1P levels also correlated with prostate-specific antigen and lymph node status. These authors also suggested that the decrease in circulating S1P during prostate cancer progression might be related to the prostate cancer-specific down-regulation of SK1 in erythrocytes and which might also account for the mechanism of cancer-induced anemia. Anemia closely follows the progression of prostate cancer. A major source for blood borne S1P is the erythrocyte and therefore circulating S1P levels are likely reduced as anemia develops and this would provide an alternative explanation of the findings.

## SPHINGOSINE KINASE 1 AS A BIOMARKER IN ER^+^ BREAST CANCER

The high tumor expression of SK1 is associated with the poor prognosis of patients with Grade 4 astrocytoma (Van Brocklyn et al., 2005). Similarly, we have reported that high tumor expression of cytoplasmic SK1 correlates with shorter disease-specific survival and recurrence time in estrogen receptor-positive (ER^+^) breast cancer patients (Long et al., 2010; Watson et al., 2010). The average survival time of these patients is reduced from 18 to 7.5 years and the time to recurrence of the disease in patients receiving tamoxifen is shortened by 8 years (Long et al., 2010). However, the oncogenic background of these patients influences the clinical outcome. Indeed, when patients were stratified according to their HER1–3 status, high cytoplasmic SK1 expression in the tumors was associated with longer disease-specific survival and recurrence times (Long et al., 2010). Thus, SK1 is protective in this HER1–3 positive cancer phenotype, thereby underscoring the need to assess the effect of SK1 on clinical outcome against a variety of other disease markers as some of these can alter the signaling functionality of SK1. In this regard, we have demonstrated that SK1 is involved in promoting the survival and migration of MCF-7 ER^+^ breast cancer cells (Long et al., 2010). However, stable enforced over-expression of HER2 increases SK1 mRNA and protein expression and activity in these cells and results in a decrease in the expression of HER2 in a negative feedback manner. This ablates both HER2 and S1P signaling linked with the migration of these cells (Long et al., 2010).

The sub-cellular localization of SK1 is also an important factor affecting clinical prognostic outcome. In this regard, the translocation of SK1 to the plasma membrane has been shown to be a critical determinant in neoplastic transformation (Pitson et al., 2005). However, our findings identify an additional novel role for the nuclear localization of SK1, which significantly shortens disease-specific survival and/or recurrence times in ER^+^ breast cancer patients (Ohotski et al., 2012a). Moreover, combinations of SK1 with other signaling proteins in the same tumor have a profound effect on clinical outcome. The analysis of these associations provides evidence for potentially new S1P-dependent signaling networks in cancer cells that can be exploited therapeutically. Thus, clinical prognostic outcome is linked with the combined high expression of nuclear SK1 and cytoplasmic phosphorylated c-RAF-1 or cytoplasmic phosphorylated AKT or nuclear ERK-1/2 expression or cytoplasmic Y416 phosphorylated SFK or LYN in the same tumor (Ohotski et al., 2012a). Some of these functional associations represent well-defined interactions; for instance, SK1 has been shown to regulate AKT and this is linked with enhanced cell proliferation and the induction of chemotherapeutic resistance in various tumors (Pyne and Pyne, 2010). SK1 expression is also higher in ER negative (ER^–^) tumors compared with ER^+^ breast tumors and this correlates with a poorer prognosis (Ruckhäberle et al., 2008), suggesting that expression levels of SK1 are associated with disease progression.

## S1P RECEPTORS AND CLINICAL PROGNOSIS IN ER^+^ BREAST CANCER

### S1P_1_ RECEPTOR

The high expression of both plasma membrane S1P_1_ receptor and cytoplasmic Y216 phosphorylated c-SRC or phosphorylated c-RAF-1 in the same tumor from ER^+^ breast cancer patients is associated with shorter recurrence time (Ohotski et al., 2012a). In addition, the high expression of both cytoplasmic S1P_1_ and ERK-1/2 or phosphorylated AKT in the same tumor is associated with shorter disease-specific survival time (Ohotski et al., 2012a). These findings suggest that the S1P_1_/AKT and S1P_1_/ERK-1/2 modules might represent spatially restricted signaling pathways in ER^+^ breast cancer patients that confer poor clinical prognosis by protecting cancer cells from apoptosis and/or promoting their growth/invasion.

### S1P_2_ RECEPTOR

In contrast with S1P_1_, high tumor nuclear expression of both c-SRC and S1P_2_ is associated with longer disease-specific survival time (Ohotski et al., 2012a). In addition, tumors with high levels of nuclear S1P_2_ receptor have significantly reduced levels of nuclear SK1, suggesting an active translocation mechanism for SK1 that is regulated by S1P_2_ and accounting for its protective action in cancer patients. The S1P_2_ receptor contains a putative nuclear localization sequence that would facilitate translocation to the nucleus and where it might function to regulate nuclear signaling linked with gene expression programs. Indeed, there are a number of reports demonstrating intra-nuclear signaling by GPCR, not least the LPA_1_ receptor that binds LPA (Waters et al., 2006).

### S1P_3_ RECEPTOR

High expression of both cytoplasmic LYN and cytoplasmic S1P_3_ or nuclear phosphorylated c-RAF-1 and nuclear S1P_3_ in the same tumor is associated with shorter disease-specific survival time (Ohotski et al., 2012a). In addition, high expression of both nuclear S1P_3_ and nuclear SK1 or cytoplasmic LYN and cytoplasmic S1P_3_ in the same tumor is associated with shorter recurrence time (Ohotski et al., 2012a). These represent entirely novel clinical and biochemical associations, which might constitute unique biomarker signatures to predict prognostic outcome in ER^+^ breast cancer patients.

## S1P RECEPTORS AND CLINICAL PROGNOSIS IN ER^–^ BREAST CANCER

**FIGURE 1:**
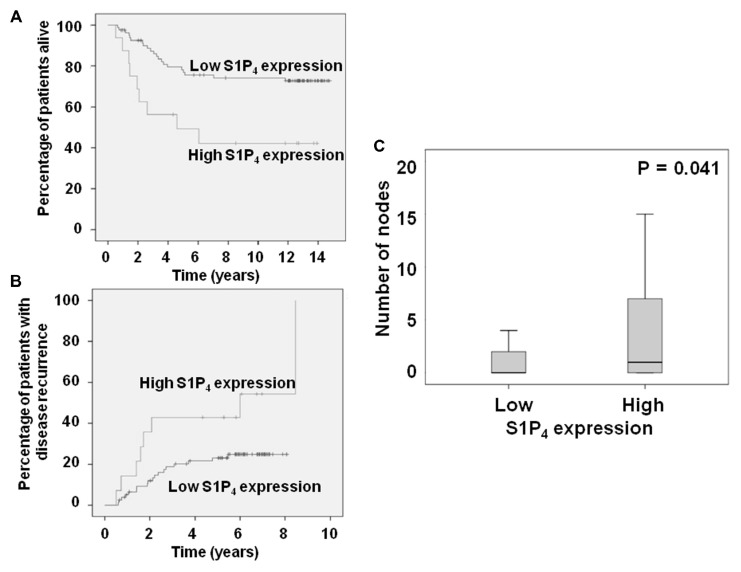
**Kaplan–Meier plots showing the effect of high cytoplasmic S1P_4_ expression on **(A)** disease-specific survival; **(B)** recurrence in ER, PgR, HER2 negative breast cancer patients**. **(C)** Box plot showing the correlation between node status and cytoplasmic S1P_4_ expression.

We have also reported that high tumor cytoplasmic S1P_4_ expression is associated with shortened disease-specific survival and recurrence times in patients with ER^–^ tumors (Ohotski et al., 2012b). We report here for the first time the stratification of these data herein to consider only patients with ER, PgR, and HER2 negative breast cancer. This analysis demonstrates that high tumor cytoplasmic S1P_4_ expression is also associated with shortened disease-specific survival and recurrence times (**Figures 1A,B**). High cytoplasmic S1P_4_ expression is also correlated with node positive status (**Figure 1C**), suggesting a role for this receptor in metastasis. Mean disease-specific survival time for patients with tumors expressing high levels of S1P_4_ was 7.3 years (*n* = 16, IQR 4.4–10.2) compared with 11.7 years (*n* = 82, IQR 10.5–12.8) for the patients with tumors expressing low levels of S1P_4_ (*p* = 0.005). Mean recurrence time for patients with tumors expressing high levels of S1P_4_ was 5.1 years (*n* = 14, IQR 3.2–7.0) compared with 6.6 years (*n* = 78, IQR 6.0–7.2) for the patients with tumors expressing low levels of S1P_4_ (*p* = 0.026). These new findings identify S1P_4_ as an important biomarker for prognostic outcome in triple negative breast cancer, and provide rationale for targeting this receptor with new chemotherapeutic anti-cancer agents.

## SK2 AND CANCER

There is also new emerging evidence for an important role of SK2 in cancer. This is exemplified by the finding that siRNA knockdown of SK2 in A498, Caki-1, or MDA-MB-231 cells reduces cell proliferation and migration/invasion and this is actually more effective than knockdown of SK1 (Gao and Smith, 2011). The knockdown of SK1 or SK2 also have differential effects on p53, p21, ERK1, ERK2, FAK, and VCAM1 indicating that SK1 and SK2 have non-overlapping functions. However, to date, there have been no studies assessing the association of SK2 expression with clinical prognosis of cancer patients.

## MONITORING BIOMARKERS AS EVIDENCE-BASED THERAPEUTICS

The major therapeutic intervention of S1P signaling in cancer focuses on: (i) inhibition of SK1 activity; (ii) antagonism of S1P_1/3_ receptors; and (iii) reduction in S1P bioavailability. Clearly, it will be important to use reliable biomarkers that provide information regarding the effectiveness of these interventions. Toward this end, we have reported that SK1 inhibitors [e.g., 2-(*p*-hydroxyanilino)-4-(*p*-chlorophenyl)thiazole, *N,N*-dimethylsphingosine, and FTY720] uniquely activate the ubiquitin-proteasomal degradation pathway to remove SK1 from breast and prostate cancer cells (Loveridge et al., 2010; Tonelli et al., 2010; Ohotski et al., 2012b) This remarkable property of SK1 inhibitors, which requires an initial inhibition of SK1 activity to activate the proteasome, indicates that it is possible to create cancer cells that are SK1 null, thereby eliminating its “oncogenic” effect. The “chemical knockdown” of SK1 reduces intracellular S1P and elevates ceramide levels, which induces apoptosis (Loveridge et al., 2010). Therefore, the “chemical knockdown” of SK1 is linked specifically with apoptosis, and may represent an important reporter for biochemical effectiveness of SK1 inhibitors in patients. The knockdown of SK1 in the tumor can be measured in biopsy samples, but monitoring in erythrocytes would be a significant advantage, although these measurements have not currently been performed.

In addition to the above, the S1P/ceramide ratio in tumors is also a biomarker for effective chemotherapeutic intervention. For instance, siRNA knockdown of SK1 expression increases the sensitivity of resistant leukemic cells to imatinib (Marfe et al., 2011) and enforced expression of SK1 increases the S1P/ceramide ratio and prevents apoptosis to imatinib (Baran et al., 2009). In addition, the S1P/ceramide ratio is reduced in response to imatinib in imatinib-sensitive LAMA84 cells, while the ratio is unaltered in imatinib-resistant cells. Finally, daunorubicin-sensitive but not insensitive leukemia cells (CML, AML, and ALL) exhibit a reduced S1P/ceramide ratio when treated with daunorubicin and sensitivity to daunorubicin in the latter is restored by inhibiting SK1 activity (Sobue et al., 2008).

Our ability to measure the clinical effectiveness of S1P receptor modifying compounds in patients by monitoring effects directly on the S1P receptor would be a major advantage. In this regard, the cytoplasmic S1P receptor might represent a surrogate marker for receptor activation. This is based on the knowledge that S1P receptor internalization is recognized as part of the process required for signal transmission regulating gene re-programing of cancer cells. Therefore, the effectiveness of S1P receptor antagonists might be linked with reduced cytoplasmic S1P receptor levels in the tumors of patients treated with these compounds.

## CONCLUSION

Clearly, the objective of S1P therapeutics is to eliminate the negative prognostic effect of S1P receptors and SK1 on disease-specific survival and recurrence in cancer patients. In addition to the expression level of these biomarkers, their activity status is also an important consideration. For instance, SK1 is activated by an ERK-1/2-specific phosphorylation of S225 in SK1 (Pitson et al., 2005). Future studies can utilize specific anti-phospho S225 SK1 antibody to determine the impact of phosphorylated SK1 on clinical prognostic significance. Moreover, specific assays that detect direct functional interaction between for instance, SK1 and an adaptor/regulatory protein using BRET and FRET technologies would enable measurement of the activation status of specific S1P-dependent signaling networks and these could then be correlated with clinical prognosis.

## Conflict of Interest Statement

The authors declare that the research was conducted in the absence of any commercial or financial relationships that could be construed as a potential conflict of interest.
